# Obstetrics—the Science and the Art

**Published:** 1849-07

**Authors:** 


### REVIEWS.

Art. V.—*Obstetrics—the Science and the Art.* By Charles D. Meigs, M. D., Professor of Midwifery and the Diseases of Women and Children, in the Jefferson Medical College; one of the Physicians to the Lying-in Department of the Pennsylvania Hospital; Vice President of the Philadelphia College of Physicians; Member of the American Philosophical Society, of the American Medical Association, etc., etc. With 120 Illustrations. Philadelphia: Lea & Blanchard. 1849.

The above is the somewhat quaint title of a work on obstetrics by the gifted Professor of that department of medicine in the Jefferson Medical College. Professor Meigs has, within a few years, produced one work after another with a rapidity truly surprising, considering the amount of professional labor daily performed by him in the great capital of which he is one of the most popular practitioners. After his treatise on midwifery, of which the present is an enlarged edition, he translated the work of Colombat de L'Isére on the diseases of females, and added to it copious notes. This was followed, in a short time, by his own work on the same subject; and this, in less than a year, by the volume before us. While he has been writing these books, comprising several thousand pages, he has not only attended to his large practice, but gone on with his lectures, and found time to contribute an occasional paper to the medical journals of the day. Such industry is worthy of all praise, and ought to be held up to the junior members of the profession for their imitation. The example of Dr. M. shows what a man by energy and determination can accomplish. Instead of making his laborious practice an excuse for neglecting his pen, he has made it the occasion of giving to the profession some of the best works produced in our country. But to return to the work before us.

It is divided into four parts, namely: the anatomy of the parts concerned in the acts of reproduction; the physiology of reproduction; the therapeutics and surgery of midwifery; the diseases of the young child.

It is not necessary to notice his account of the anatomy of the bony pelvis, nor shall we follow him through his description of the mechanism of labor.

In his third chapter he describes the form, size, sutures, and fontanels of the fetal head. Dr. M. holds that, rigorously speaking, there are but two presentations in midwifery "one of the head, the other of the pelvis." He regards presentations of the shoulder, arm, face, and forehead, as deviations from the vertex; those of the feet and knees as deviations from the pelvis.

Chapter fourth treats of the genitalia, and in the fifth chapter he gives an account of the anatomy and physiology of the ovaries, Graafian vesicles, and of the ova therein contained. In some of the opinions expressed concerning the nature of the corpus luteum the author is at variance with other microscopists, and renewed observations are necessary to settle the question as to its precise function.

As to fecundation, he thinks, with Pouchet, that it cannot take place in the ovaria, and yet he admits that it has been shown by Bischoff and Barry that the semen does ascend as high. We are not able to see how he can reconcile these contradictory statements. If the semen reaches the ovary, what objection is there to the "antiquated notions as to ovaric fecundation?" He is surprised that any one should cling to what he says Pouchet "has shown to be an impossibility." His readers, we think, will feel surprise that, with all the facts before him, many of which he adduces in his work, he should doubt that ovarian fecundation and ovarian pregnancy are both possible. Too many well authenticated cases are on record to admit any longer of doubt on the subject.

Dr. Meigs is devoted to the modern theory of menstruation, of which he gives a satisfactory account in his sixth chapter. According to this theory, ova at the age of puberty are matured in the Graafian vesicles, and continue to ripen until the female ceases to be capable of childbearing. They ripen periodically, and in women, for the most part, once in four weeks. During the last days of their development, the uterus becomes congested, increases in weight, and sinks somewhat lower in the pelvis. From this state of engorgement it is relieved by means of the menstrual hemorrhage.

This is a captivating view of the subject, and one could wish that it were divested of all difficulties. But it is met by serious objections. Dr. Ritchie, for example, asserts confidently, that maturation of ova takes place previously to puberty. He even goes so far as to say that they are matured during childhood. If this assertion is to be credited, it is evident that the cause assigned for the catamenia cannot be the true one.

It has been further objected, that ova are matured and expelled at other than stated periods. While Bischoff, Pouchet, and Raciborski, in all their examinations of females dying about the catamenial epoch found evidences of the maturation and expulsion of ova, Dr. Ashwell asserts that he found no such evidences in the ovaries of three females examined by him during the menstrual flux. Others maintain that ovulation occurs, in the human female, at all periods, as well between as during the menstrual epochs; and this doctrine is in harmony with the fact that women seem to be susceptible of impregnation at all times of the intermenstrual period. Still, we are not prepared to abandon so easily a doctrine which has so much beautiful analogy to support it. We are inclined, despite the many facts urged in opposition to it, to hold to its validity. It is one of the theories which, if not true, one feels ought to be true. Why there should be this irregularity in the economy of nature—why in all the lower animals ovulation should be periodical, and only constant in the human female, it is not easy to perceive a reason. As in the inferior races there is a limit beyond which conception is impossible, and this is rigidly restricted to the few days or hours during which the female is in heat, so analogy would lead us to suppose that a woman cannot conceive after the lapse of a given period. Dr. Meigs is willing to believe with Pouchet, that the capacity is limited to the twelfth day; but, as he remarks, "time, and opportunity to observe, can alone settle this point." For the present we avow our inclination to embrace the new theory.

The chapter on amenorrhea is practical, and abounds in judicious views. The author indicates correctly the cause of the great empiricism shown in the treatment of the affection. He shows that it has grown out of a bad pathology. The proximate cause of amenorrhea has not been thoroughly investigated as, in every case, it is necessary to do. The state of the patient's health has not been carefully studied. If a woman's constitution "be brought into healthful play in all other regards, she will not be vicious or disordered in this instance, of menstruation." A woman is not unhealthy because she fails to menstruate, "but she fails to menstruate because she is unhealthy." This, we believe, is the true doctrine on the subject. The cure, therefore, is to be sought in that act, or that article of the materia medica, which obviates the cause. Specific emmenagogues will not answer the purpose. That which would afford relief in one instance, in another would aggravate and confirm the disease.

The causes of amenorrhea are numerous; but the most productive, in the views of our author, is the "cessation of the force by which ovarian vesicles are evolved and matured." The vital action which brings the ova to maturity being destroyed, the catamenial hemorrhage, which is but a sign of the menstrual act, cannot appear. "The reproductive is a complementary force, and menstruation is a sign of the active state of that force." The amenorrheal girl is generally anemic; the system in anemia is not capable of developing the ovarian vesicles. The system of cure, in her case, is evident. It consists in the use of all the agents which correct anemia, and give tone to the economy. Aloes, especially, is indicated as an aperient, because it determines to the pelvic viscera. For improving the hematosis there is no article equal to iron.

Of the ferruginous preparations, our author prefers the impalpable powder of the metal, produced by passing a stream of hydrogen gas over the peroxide of iron heated to redness in a porcelain tube. He exhibits this in the form of pills weighing two grains, one soon after each meal. We agree with him, not only that iron is by far the most effectual remedy in all cases of amenorrhea in anemic subjects, but that, since the metal itself is the therapeutical principle, there is little advantage in exhibiting it in combination with any particular acid. We have found the peroxide a reliable agent in the anemic diathesis, and have also used the iodide with great advantage in such cases.

The next chapter treats of pregnancy, and at the beginning, the author "fully acknowledges his ignorance of the essential nature of fecundation." The germ is undoubtedly produced by the female, and it is nearly certain that this is a cell, as it is probable that the spermzoon is of the same nature. But which of these cells has the prëeminence, or is the true germ; which "is the primal solid in the generic or fecundative processes," are questions which no physiologist has yet answered, if, indeed, the mystery is ever to be understood.

"If conception take place in the womb, it is pregnancy —if out of the womb, it is extra-uterine pregnancy—in the Fallopian tube, tubal pregnancy—in the ovarium, ovaric pregnancy." Yet our author had expressed his surprise, in a former chapter, "that many able and distinguished writers still cling to the antiquated notions as to the ovaric fecundation, which M. Pouchet has shown to be an impossibility." As we have already remarked, we cannot see how these passages are to be reconciled. We wonder that they should have occurred in the same work.

The nearer to the close of the menstrua, the more likely is the congress of the sexes to result in fecundation; but conception does not necessarily follow; for the fecundated ovum may not effect its mesenteric attachment, but be discharged from the uterus and lost. When it becomes fixed, conception properly commences.

Dr. Meigs is convinced that ovulation occasionally precedes the menstrual flow, but that in the majority of cases it absolutely coincides, in period of time, with the first appearance of the hemorrhage. He very properly repudiates the notion, that it is connected, in any way, with the aphrodisiac orgasm, since the ovulative paroxysm is as decided in the virgin as in the married woman.

Referring, again, to the question, at what point does "the encounter of the sexual elements take place," he says, "we do know that it may, and sometimes does, occur in the Fallopian tube." He thinks it possible that it may likewise occur within the womb itself, and suggests that the accidents of placenta previa may be "the results of a late fecundation, which admits of the fixation of the product upon the os uteri internum."

The author alludes, in this chapter, to the vomiting which often constitutes so troublesome an attendant upon pregnancy. In one case, this symptom persisted up to the time of confinement, and proved a serious hindrance to the progress of labor. The patient was a primipara, and although, as a general rule, he regards it as bad practice to rupture the membranes in such labors, he adopted the practice in this instance, and from the moment that it was done, "the nine-months' vomiting ceased and returned no more." The following case shows that, however distressing this concomitant may be, its consequences are not necessarily serious even where the danger seems to be greatest. He says:

"I attended a lady in Spruce street, a few years since, who, during nearly three consecutive months, appeared to vomit up every particle of her ingesta. It was her own opinion, coincided in by her friends and attendants, that the total amount of all her food and drinks returned very soon after they were swallowed. Although she felt much weakened, I could not perceive that, under this process, she lost her flesh, and in the end, she gave birth to a healthy daughter. It is apparent that she must have been nourished during this time—but the manner, and the quantity, have remained ever since a mystery that I cannot explain."

Many of these cases are curable by exercise, either on foot or in a carriage. In perhaps all, relief is derived from regulating the bowels, which are generally constipated. Tonics and alkalies are the remedies most generally resorted to, and the latter are nearly indispensable as correctives of acidity. Dr. Meigs mentions a method of treating cases in which vomiting comes on early in the morning and ceases about noon, the rationale of which is not very intelligible. He assures us, however, that he has found the course successful; it is as follows:

"Let a cup of coffee, with a toast, be brought to the bedside at the earliest morning hour. The patient should be called from her sleep to take this preliminary breakfast, without rising from bed. As soon as it is taken let her lie down to sleep again, if possible."

Dr. Meigs dissents from the Hunterian doctrine with respect to the placenta, and contends, with Velpeau, in opposition to Professor Owen, that no part of this appendage belongs to the uterus, but that it is entirely fetal. He does not believe that there is a vascular connection between the placenta and the uterus. Many examinations have been made by him, and in none could he trace vessels passing from one to the other, and his conclusion from the whole is, that the placenta is not more adherent to the womb, "than is the peel of a perfectly ripe orange to the fruit." At the same time, he admits that there may be a strong argument in favor of the other view of the subject, and he states it at some length. It is very certain that no doctrine which had not many arguments to support it could ever have gained the advocacy of such physiologists as the Hunters and Prof. Owen; but it does not therefore follow, that theirs is necessarily the true doctrine. In such questions mere authority is nothing. These accurate observers may have been mistaken. At all events, as remarked by Dr. Meigs, one must be permitted to see with his own eyes.

A clear description is given of the fetal circulation, after which our author goes on to remark as follows:

"The branchial apparatus above described, suffices in all the mammals and birds to communicate to the constitution of the embryo the requisite amount of oxygen; but it ought to be observed that that amount is small, indeed, when compared with the freeness of the endowment vouchsafed to a state of respiratory existence. The embryo requires no more than what suffices to oxygenate its fluids and solids to the extent of provoking an active nutrition and impart a power of gentle and rare muscular motion—for the fetus in utero may be regarded as torpid, and as approaching in torpidity to the state of hybernating animals. To cut off even this slender supply is to ensure its destruction. Now, inasmuch as the placental blood, entering in at the umbilicus, passing by the ductus venosus to the inferior cava, along that tube to the auricle, and through the foramen Botalli to the left auricle, left ventricle, aorta, carotids and vertebrals to the brain, takes the only possible route from the placenta to the brain; it is clear that if, before the birth, the foramen ovale should be closed, no oxygen could possibly reach the brain; but oxygen in the brain is essential to the evolution of biotic force. When, therefore, no oxygen reaches the brain, the brain evolves no nerve force, and the patient being asphyxiated dies. The law, then, is that the fetus is born with an open foramen ovale, which becomes closed after birth, generally within three or four days, often in ten or twelve days, not rarely about the twentieth day, and sometimes never.

"I have said that the child's foramen Botalli remains open during the whole uterine life; but the student ought to observe that it is always covered by its valve, lying upon the left side of the septum—a valve so light and delicate as to be transparent, and so beautifully arranged as to enable it to cover the operculum in the most perfect manner. The weight of a drop of blood, resting on its right side, might lift, as a drop on its left surface might shut it down. The normal direction of the current through the foramen, keeps it open in the fetal heart.—When, therefore, after the child is born, the two auricles act at the same time, in equal times and with the same intensity, the valve is pressed upon the operculum to cut off the fetal route, and compel the whole of the right auricular torrent to pass to the right ventricle. If the left auricle should be the strongest, the earliest, and the longest to contract, it is impossible that any black blood should come into it. If, on the other hand, the right auricle should, after the birth, contract sooner, longer, and more energetically than the left, the valve of Botalli would be lifted, and the black blood of the venous system, instead of returning by the pulmonary ventricle and artery to the lungs, would pass to the left auricle, ventricle, and aorta to inundate the neurine of the brain with its carboniferous stream, which, wholly incapable of exciting any biotic force in the brain, would be cyanosis—asphyxia—death. When the nervous mass ceases to act, the whole constitution is dead. It always ceases to act where there is no oxygen."

The circulation of the fetus, and that phenomenon which frequently presents itself in the new born babe, cyanosis, have been fruitful topics of discussion. We have quoted extensively from our author on this point, both because we like his views, and because the extract given affords a good specimen of the physiology in which his work abounds. The whole elaborate chapter on pregnancy will be read by the student with the deepest interest. Some of the diseases to which the child in utero is liable are mentioned in this chapter, and various kinds of monstrosity are illustrated by wood-cuts. Some of these afford, as remarked by Dr. M., curious subjects of reflection—" the individuality or duality of a creature with one head and two bodies, or with two heads and one body?" These are questions for the metaphysician. We quote a few paragraphs on this singular subject.

" Doubtless, it is not possible, in Teratology, to suppose that half of one child should sink into and be totally lost in half of another child, thus making out of two independent personal identities a single one. In nature, the union must take place from the liver upwards only, or from the liver downwards only ; whence, it cannot happen that the whole right symmetrical half of the left twin should be sunk in the left symmetrical half of the right twin. We may therefore expect to meet with cephalodym or hepatodym or pelvidym, and not with such a union of two personal identities as would be to fulfil the ancient fable of the union of Salmacis and her lover.

"All such fusions imply loss, not gain of substance; or monstrosity by default, and not monstrosity by excess.—If a child is born with six fingers on either or each hand, or six toes on either or each foot, it presents a case of excess of development or monstrosity by excess; and the samples of five-legged calves, &c., that are commonly met with, are cases of monstrosity by excess.

"There was a singular example of cephalodym here some four years ago: it was a healthy pig with one head, two fore legs, and two bodies, with four hind legs. It was a remarkable fact that the genitalia of this creature were not ruled by a common influence of its nervous systhem; when the animal was in heat, it was either as to the genitalia of the right or those of the left trunk; but they were not observed to be in heat or rut at the same time, one trunk appearing to become the subject of the periodical excitement about ten days after the other had ceased to be so. What was the condition of personal identity in this monster!"

In regard to the duration of pregnancy, our author entertains views that most of his brethren will consider latitudinarian—he believes that it may "endure even beyond twelve months." And analogy, it must be owned, is favorable to this opinion. The term of incubation of the barn-door fowl varies two or three days, and the term of utero-gestation in our domestic animals varies weeks and even months, according to the testimony of several observers. Brugnone found that the foaling of fifty-five mares took place at periods varying from ten to thirteen months. In one, it occurred before the completion of the tenth month, and in several it was delayed to within a few days of twelve months. Two mares foaled in 330 days, four in 336, five in 343, two in 352, one in 369, and one in 389—a difference between the most precocious and most protracted gestation of two months and a half. Tessier found that in the gestation of 200 mares there was a latitude of 83 days. Grille found the difference 93 days, and others have found it as great as 97 days. In cows, a latitude of 23 days has been observed. These things being so with regard to the inferior animals, there seems to be no good reason why we should doubt the possibility of such variations in the human female.

The uterine muscles, in our author's belief, cannot be traced by the scalpel, however little their existence can be doubted by any one who has had occasion to attempt turning a child in a powerful womb. He has no faith in any of the representations given by the several writers who have professed to describe them. The discrepancies of these authors are such as to convince any one that the arrangement is not understood.

The prominent disorders and accidents incident to utero-gestation are alluded to in the close of this chapter, among which are obliquity, pressure of the womb on the vessels, anemia gravidi, moles, hydatid degeneration of the ovum, physometra, hydrometra, abortion, prolapsus, retroversion, and extra-uterine pregnancy.

The opening to the third part, or the therapeutics and surgery of midwifery, is in a very graceful and pleasing style. The young accoucheur is exhorted to consider seriously the delicate relations he is about to assume, in which so much tact, discretion, and good sense are necessary to success. Above all, he is cautioned against revealing professional secrets, and against that tone of familiarity to his patients which some affect, but which is so liable to be construed into impertinence, and is sure to offend the refined of the sex. The following incident is related as illustrative of the baneful effects of babbling:

"The great Caliph Al-Mamun, as we are informed by Abul-Pharajius, in his History of the Dynasties, was a friend of science, and he exhibited his patronage, by fostering many learned men, among whom were some of our own profession.

"Among other of his medical favorites, was John Ocularius, whose duty it was to visit the Commander of the Faithful every day, and that in his most private apartment, alone.

"The caliph gave him great honor, and allowed him a monthly stipend of one thousand gold sequins for his services.

"Upon one occasion, as the physician came out of his master's apartment, in passing through an ante-room, he was asked by one of the servants, 'What is the caliph doing?' 'He is sleeping,' was the reply. But, unhappily, this reply was communicated to the successor of Mahomet, whereupon the culprit was sent for, and brought before the chief of Islam. 'What!' said he to the offender, 'have I employed you as my physician, and admitted you to my intimacy, in order that you should report to my servants as to my private occupations? Go out of my house.'"

The unfortunate physician, in relating this story, to ac count for his disgrace, added that Al-Mamun would never afterwards admit him into his presence; which, Dr. M. declares, "was the just punishment of a professional indiscretion;" and he adds—

"Let the student reflect upon the punishment deserved by those who babble the concerns of families or individuals. John Ocularius was turned out of the court of Al-Mamun for merely saying that his master was asleep!"

Labor, in the view of our author, has for its cause "the inability of the womb, in any given case, to bear further distention." It has its beginning "in a necessity of the uterine constitution, and not from any ascertained degree of development of the child, which, whether large or small, is most likely to be born two hundred and eighty days after the last catamenial period of the mother; but may not be born until three hundred, or even more days have elapsed." Whatever be the secret forces on which the commencement of labor depends, and whether or not we shall ever be able to unravel them, we behold in the development of the gravid uterus and its contents, as remarked by Dr. M., "a wonderful adaptation of parts to the purposes they are destined to fulfil;" for if the child continued to grow, its expulsion, in the end, would be impossible. As now arranged, the womb, by a simple law, refuses "to yield any further than is sufficient to allow the child to acquire a certain degree of magnitude and vigor, essential for its respiratory life, but not too considerable to prevent its birth from taking place." And, as added by Dr. M., "this perhaps is, after all, a sufficient solution of the problem."

The pains of labor are owing to the sensibility, not of the expelling, but of the resisting organs—the cervix and os uteri, the vagina and external parts. Dr. M. remarks that

"The actions of the woman indicate pretty clearly, to the practised eye, the state of advancement of the process. Antecedently to the exit of the head from the os uteri, or its deep insertion into that circle, the voluntary efforts of the patient are confined to a violent grasping of things with her hands. She generally seizes the hand of a bystander, and squeezes it violently, or endeavors to twist or wring it, not pull it. Such an action always indicates a grinder, or a pain of dilatation; but when an expulsive effort takes place, she not only grasps with all her force, but she pulls at anything in her reach; so that an experienced accoucheur generally can decide, upon entering the chamber during a pain, that the dilatation is or is not completed, by observing whether the patient merely squeezes or presses the hands of her assistants, or, on the other hand, whether she pulls them with great violence."

The phenomena of a case of labor suggest to the mind of our author "a perfect example of the state of hysteria." The process, as he expresses it, "is a pure specimen of the local action and constitutional influence displayed by the childbearing organs, and the whole of what Wigand calls the generation sphere."

The conduct of a labor forms the subject of the tenth chapter, in speaking of which our author remarks, that he "cannot well eschew to say something upon the employment of those anesthetic agents, whose recent irruption into the domain of medicine and surgery has been so sudden, violent, and overbearing." He avows that it would have been more consonant with his taste as well as with his views of medical duty, to have avoided any notice of those agents. We doubt not the subject is distasteful to him. He finds himself almost alone in his opposition to the practice, and his position cannot be otherwise than uncomfortable. The letter of his friend, Prof. Simpson, published not long since in the Medical Examiner, would disincline almost any one holding Dr. M.'s views, to the discussion of anesthesia.

It is a somewhat curious circumstance, as stated by Dr. Meigs, that in the city of Philadelphia, "the use of ether and chloroform in surgery and midwifery, has made no real progress," while the practice has become general in nearly every other great city in the United State, and in Europe. Philadelphia alone stands out against anesthesia. Whether the practitioners of that metropolis are wiser than their brethren, or only excessively cautious, is a question to be decided by a longer trial of etherization. But certain it is, that the current of professional opinion is now powerfully against them, and although it may be true that in that community "fewer women were etherized in the last than in the first six months of 1848," such cannot be said of other communities. The practice everywhere else is gaining ground, and we find the Committee on Obstetrics reporting to the Medical Association that the question, at present, is not whether anesthetics may or may not be safely administered, but whether they *can be rightfully withheld* in the practice of midwifery.—With all the testimony of the last eighteen months before him, we are not surprised that Prof. Meigs feels some distaste to the subject. Indeed, it would be strange if he felt perfectly easy in his position. If the judgment of Prof. Simpson, which is also the conviction of a multitude of other observers, be a correct one, that chloroform and ether, while they render labor painless, also greatly increase the chances of the patient's recovery, the accoucheur who refuses to employ them is doing his patients great injustice; and the bare suspicion of such an error would create anxiety in the mind of a sensitive and humane practitioner. We do not conceive the objection a sufficient one, that anesthetics have occasionally proved seriously deleterious, even to the extent of suddenly destroying life. The same argument might be urged against all the potent articles of the materia medica. Opium, antimony, mercury, even quinine, has serious allegations to answer, and the physician who sets out resolved to employ no medicines capable of doing mischief, must discard these time-honored remedies from the roll of his therapeutic agents. But, instead of meeting, ourselves, the objections of Prof. Meigs to the use of anesthetics in midwifery, we prefer, (and we believe our readers will like our choice) giving some extracts from the masterly reply of Prof. Simpson. Prof. M. objects to anesthesia in deliveries requiring "chirurgical intervention," and especially in forceps operations, on the ground that the sensations of the patient afford us our best guide for the introduction of the instrument. To which Prof. S. replies:

"In answer to this novel objection, you will excuse me when I say it, (for I say it most conscientiously,) that I think every man who ventures to use the forceps in any midwifery case, ought to know the anatomy of the parts implicated, a thousand fold better than you here presuppose. You would have the accoucheur guide his instrument, not so much by his own anatomical knowledge, as by the feelings and sensations of his patient. In this, as in other points, relative to any novel question in practice, we can often, it appears to me, best perceive the soundness or unsoundness of our views upon it by considering and contrasting them with our established views on other analogous questions, regarding which, the opinions of the profession have been long fixed and determined.—Now, what would the surgical world, at this time of day, think of an operator, who, in making a ligature of a large artery, such as the humeral, placed his chance of discriminating the attendant nerve from the bloodvessel which he is in search of and which he wished to tie, by appealing, not to his own anatomical knowledge, but to the feelings of his patient, as he touched the suspected structures. 'Does it hurt you, yes or no." Would our surgical brethren not denounce and decry the capabilities of any man who, in operating, should require to have recourse to such imperfect and incompetent diagnosis?—Would it be right and moral in a surgeon to deny to his patients the advantages of anesthesia, in order that their sensations and sufferings should make up for his want of anatomical and operative knowledge.

But in saying this, do not, I pray you, for one moment suppose that I fancy that the argument which you adduce, betrays any want whatever, of the highest degree of operative skill on your part. Nothing could be further from my thoughts. And, to confess the truth, I do sincerely believe, that you, yourself, while using the forceps, do not require to have recourse to any such rule as you here propound; and that in fact, the rule itself, and the objection to anesthesia in midwifery which it contains, is an *afterthought* on your part, which has only sprung up since the practice of anesthesia was proposed. For, in looking over the excellent precepts which you have given relative to the use of the forceps in the valuable work on midwifery, which you published a few years ago, (viz., the Philadelphia Practice of Midwifery,) I find no trace or mention whatever of such a rule as you have quoted above in your letter to me. If that rule really formed, as you now state, the 'safest and most trustworthy' guide in the operation, you would certainly have at least noticed it, or alluded to it in some way. In the precepts which you laid down in your work, you would assuredly not have forgotten that one rule, which, you say, is worth a 'thousand other dogmas and precepts.' And I think it would have been only the more incumbent upon you to have mentioned it, seeing that all other authors omit the notice of it.

"I feel assured that when you come to reconsider 'dispassionately' your opinions regarding the non-employment of anesthesia in operative midwifery, you will alter those opinions. And when you come to employ anesthesia in actual practice, in cases in which the forceps are used, you will find that instead of impeding the application of the instruments, the anesthetic state very greatly facilitates it. It enables you to guide the forceps far more safely to their destination, because it enables you, without any pain to the patient, to introduce your fingers for this purpose, far more deeply between the head and maternal structures than you could do if the patient were awake and in her usual sensitive state. You, yourself, state, in your published work on midwifery, that care should be 'taken to direct the point (of the forceps) by the two fingers as far as they can reach.' (p. 300.) 'If (you again observe,) any difficulty occurs in getting the second blade forwards enough, the two left fingers that are guiding it will serve to guide it edgeways into the proper position.' Now, the state of anesthesia, I repeat, gives you (as I have several times found,) the power of fulfilling these and other important rules, to an extent that never can be attained without it, and I am sure you will find them worth any 'thousand dogmas and precepts,' derivable from the mere sensations of the patient."

Again; Prof. M. objects to the use of anesthetics in natural labors, because he holds that the pain in such labors ought not to be annulled, and that it is calculated to promote the safety of the operation—that, in other words it is "a most desirable, salutary, and conservative manifestation of life-force." To this Prof. S. replies as follows—

"In the above expressions you make no distinction between the two separate and distinct elements of which a so-called labor-pain consists, viz.: 1, the contractions of the uterus, and 2, the sensations of pain resulting from these contractions. If you apply the language I have quoted to the first of these elements, the uterine contractions, (and which contractions are not annulled by anesthetics,) I decidedly and entirely agree with you. If you apply it, however, to the sensations of pain produced by the uterine, contractions (and which sensations are annulled by anesthetics,) I most decidedly and entirely dissent from youropinion."

"The muscular contractions of the uterus form, you say, the 'essential element' of labor. In that opinion you and I are at one; and further, I quite agree, that this cannot be safely 'eschewed and obviated' in natural labor, nor are they 'eschewed and obviated' under the proper use of chloroform.

"But the pain, the second element, is a non-essential element in the process. It is non-essential, because, 1st, labor, that is, the uterine contractions, are occasionally, though very rarely in the course of practice, seen to accomplish the full expulsion of the child with little or no pain. 2d. In whole tribes of the human race, as in some of the black tribes, little or no pain seems to be endured, if we may believe various authorities; and 3d, hundreds of women have, during the last year, been delivered with perfect safety, but without any pain, while placed under the influence of anesthetic agents.

"I hold the pain to be non-essential, and I protest against the truth of your opinion that 'the pain of a natural labor is a state not by all possible means to be eschewed and obviated.' On the contrary, I maintain that we omit and forego a weighty part of our professional duties whenever we forget the axiom of Bacon, that 'it is the office of a physician, not only to restore health, but to mitigate pain and dolours.' And as medical men we are called upon to mitigate and remove those 'pangs and agonies of travail, (as you term them,) which, in degree, are, in your own language, 'absolutely indescribable, and comparable to no other pain,'—pain, for which there is 'no other name but agony.'

"In your practice, you, like other medical men, constantly use measures to mitigate and relieve the pains of headache, of colic, of sciatica, of pleurodyne, of gout, of rheumatism, and all the other innumerable 'dolours' that flesh is heir to. Like other physicians, you deem it, I doubt not, your duty to wield the powers of your art, in order to free those who submit themselves to your medical care from these and other similar sufferings. But, if it is right for you to relieve and remove these pains, why is it not right for you also to relieve and remove the pains accompanying the act of parturition? I cannot see on what principle of philosophy, or morality or humanity, a physician should consider it his duty to alleviate and abolish, when possible, the many minor pains to which his patients are subject; and yet should consider it improper to alleviate and abolish, when possible, pains of so aggravated a character that, in your own language, they are 'absolutely indescribable, and comparable to no other pains,"—pains for which there is 'no other name but agony.'

"3. *You object to anesthesia in natural labor because you deem the pain of natural labor 'a physiological pain.'*

"'The sensation of pain in labor is (you observe) a physiological relation of the power or force,' and 'to be in natural labor is the culminating point of the female somatic forces.'

"Now, for the reasons that I have already stated, I entirely doubt if we should look upon the severe sensations of pain endured by our patients as truly 'physiological,' for, as I have just stated, they are not essential to the mechanism and completion of the process in the white races of mankind, and they are absent to a great degree in the black. The severity of them could, I think, be easily proved to be the result of civilization, and, as I believe, of that increased size of the infantile head which results from civilization. Parturition is always physiological in its object, but not in some of the phenomena and peculiarities which attend upon it in civilized life.

"But waiving this point or the discussion of it, let me state that even if I allowed all the intense pain of parturition to be 'physiological pains,' I cannot conceive that to be any adequate reason for our not relieving women from the endurance of them. Because nature has fashioned any particular physiological function in any particular manner, that, I opine, is no reason why the science and art of civilized life should not, when possible, alter and amend its workings. If it were improper for us, for instance to intermeddle with the functions of the hair of the head, or of the skin generally, then all hats and other coverings for the scalp, all clothing and coverings for the body, should be at once abandoned and unconditionally condemned. If it were improper for us to alter and amend the functions of the eye, then all optical glasses, the telescope, the microscope, &c. must be thrown aside. And indeed not later than the 17th century, it was held and argued so in England. For in his history of the first beginning of the Royal Society of London, Sprat tells us that it was generally believed that this new experimental philosophy (namely the philosophical papers laid before the Society,) was subversive of the Christian faith, and many, he adds, mortally hated the newly invented glasses, the telescope and the microscope, as *atheistical inventions*, which perverted our organs of sight, and made everything appear in a new and false light, (D'Israeli.)

"You argue as if supposing that we should not use means to eschew the pain of parturition, because that pain is physiological. When Columbus first discovered your mighty American continent, a large portion of the inhabitants were unprovided with any kind of dress or covering. 'To most of them,' says Robertson, 'nature had not even suggested any idea of impropriety in being altogether uncovered." And I do think that men living in such a state could, against the fashion of dressing, use with far greater propriety and consistency than you or I, your own argument against anesthetics in labor. Chloroform and ether should not be used in labor, (you argue,) because the pain against which they protect us is natural and physiological. No kinds of clothing or dress should be used, (the original Americans might have equally argued,) because the cold and heat against which they protect us are natural and physiological.

"I have a letter lying before me on the subject of anesthetics in midwifery, by a very highly and very justly esteemed teacher of midwifery in Dublin. 'I do not (he writes,) believe that any one in Dublin has as yet used ether in midwifery—the feeling is very strong against its use in ordinary cases, and merely to avert the ordinary amount of pain which the Almighty has seen fit—and most wisely we cannot doubt—to allot to natural labor, and in this feeling I heartily and entirely concur.'

The argument thus used, and so very well expressed by my Irish correspondent, is one which has often been adduced and repeated during the course of the past year. Some minds at first gave immense weight and importance to it. For my own part, I must confess that I never could view it as containing any great force. Look at it as applied to any other practice which happens to be sufficiently old and established: and then we will see it in its true import. Supposing, for example, it referred to the first introduction of carriages into use: It would then read thus,—I do not believe that any one in Dublin has as yet used a carriage in locomotion; the feeling is very strong against its use in ordinary progression, and merely to avert the ordinary amount of fatigue which the Almighty has seen fit—and most wisely we cannot doubt—to allot to natural walking, and in this feeling I heartily and entirely concur.

"Nay, this frequently repeated argument against new innovations becomes not only, I think, ridiculous, but really almost irreverent when we look far backwards into the march of civilization, and apply it to any practices that are so very long established as to be very antiquated and with which, therefore, the human mind has been long and intimately familiarized. Some one, but who I cannot pretend to say, no doubt first introduced the practice of wearing hats or bonnets or some covering for the head.—Suppose this practice, however, strongly resisted as doubtlessly it was at first, then the argument of my Dublin friend against this innovation would read somewhat as follows:—I do not believe that any one in Dublin has as yet used a hat to protect his head; the feeling here is very strong against its use in ordinary weather, and merely to avert the ordinary amount of wetting and cold which the Almighty has seen fit—and most wisely we cannot doubt—to allot to mankind, and in this feeling I heartily and entirely concur."

Prof. S. continues his reply in the following style—

"You maintain that 'labor is the culminating point of the female somatic forces.' One of the most illustrious Presidents of your great American republic—Thomas Jefferson—makes in his memoir a remark of precisely the same import, regarding walking or progression. He des cribes the act of walking, but not exactly in the same words, as the kind of " culminating point of the human somatic forces.'

" Few or none, perhaps, will question the abstract truth of Jefferson's observations on this point. But, because walking or progression is a 'physiological' function, and the practice of it is reputed salutary, would this be with you a proper and sufficient reason for never setting aside or superseding in any way this 'physiological' state, in the same way as you insist, on the same grounds, that the physiological pain of labor should not be set aside or superseded? Because progression is a natural condition, would this be any adequate reason for your medical advisers adopting your own arguments against anesthesia in midwifery, and insisting upon this, that, the next time you travelled from your own city of Philadelphia to the cities of Baltimore or New York, you should walk the distance on foot instead of traveling it by railway or other conveyance? What opinion would you form of the judgment of any medical adviser to whom you entrusted your own health, if, on going next time to the New York or Baltimore railway station, he should gravely and solemnly repeat to you as his patient what you tell your midwifery patients, and, in your own language advise you to try to accomplish the intended journey on foot, as (to quote your own words,) 'a desirable, salutary, and conservative manifestation of life-force?' And yet this would really be nothing more than making your *argumentum ad fæminam* an *argumentum ad hominem.*

" You state, in a passage which I have already quoted, that even the agony accompanying instrumental delivery by the forceps, is a 'physiological pain.' I do not, I confess, see why the sufferings attendant on the use of the forceps, when the head is impeded by any cause of obstruction, should be regarded as a physiological pain, any more than the suffering attending the use of the catheter in obstruction from the prostate gland, or other morbid conditions of the urethra should be regarded as a 'physiological pain.' They are both operations intended to remove the natural contents of the respective viscera, when their operative removal becomes necessary.

But let us waive this point, and return again to the analogy between the functions of progression and parturition. Suppose that you plead with your medical advisers that, instead of insisting on your going on foot, they should allow you for once to take advantage of artificial assistance, and proceed on your journey from Philadelphia to Baltimore, or New York, by railway, because you were unable to walk the distance, in consequence of being incapacitated by a rheumatic knee, or a sprained ankle, or an inflamed or blistered toe, and they replied to you that you should not care for this, but still proceed and suffer, because the pain you might thus suffer, was (to use again your own language,) still only a 'physiological pain.' Would that argument be any adequate philosophic consolation under the endurance of your suffering? Or would you not laugh at the logic of your medical adviser, and take your seat in the railway car in spite of his doctrine? And I have a fancy, that betimes, in midwifery, patients *will* have to adopt exactly the same line of practice under the analogous circumstances, and think, and act, too, in the same way."

Our space is taken up, and we must here close, for the present, our examination of this admirable work; for admirable it is, despite the errors of doctrine and defects of style, examples of which it would not be difficult to multiply. No reader, we venture to say, will lay the volume down without admiration for the learning and talents of its author. But there is one class—a most interesting class—who, we fear, will close it with feelings of disappointment. We mean the ladies. When they, who have become familiar with the easy style of Dewees, come to turn over these pages, they will be confounded. We are not without fears that not a few readers of the other sex will occasionally find themselves embarrassed. We could wish to see the work somewhat amended in this respect. An abler volume, upon the whole, we do not hope soon to see.

